# Perimesencephalic Subarachnoid Hemorrhage Is Not Always a Benign Condition: Hemorrhage Volume as a Predictor for Complications and Clinical Outcome

**DOI:** 10.3390/biomedicines13051061

**Published:** 2025-04-27

**Authors:** Emily Hoffmann, Công Duy Bùi, Alexandra Valls Chavarria, Michael Müther, Markus Holling, Manfred Musigmann, Max Masthoff, Mostafa Ergawy, Tobias D. Faizy, Christian Paul Stracke, Hermann Krähling, Burak Han Akkurt

**Affiliations:** 1 Clinic of Radiology, University of Münster, 48149 Münster, Germany; 2 Department of Neurosurgery, University of Münster, 48149 Münster, Germany; 3 Clinic of Radiology, Division of Interventional Neuroradiology, University of Münster, 48149 Münster, Germany

**Keywords:** perimesencephalic, subarachnoid, hemorrhage, vasospasm, hydrocephalus, delayed cerebral ischemia, clinical outcome, hemorrhage volume, imaging

## Abstract

**Objective:** The benign nature of perimesencephalic subarachnoid hemorrhage (pmSAH) can be challenged by the occurrence of complications. Given the limited prognostic value of established clinical parameters for the development of complications in patients with pmSAH, this study evaluates the potential of volumetric hemorrhage quantification for risk assessment and the evaluation of the clinical outcome. **Material and Methods**: In this retrospective single-center study, we analyzed all consecutive patients diagnosed with pmSAH between 2010 and 2023 at a tertiary care academic medical center in Germany. The volumetric quantification of the hemorrhage in cm^3^ was performed using non-contrast CT imaging. The occurrence of clinical complications, including hydrocephalus, vasospasm, and delayed cerebral ischemia (DCI), were assessed. Clinical outcomes were determined by the Glasgow Outcome Scale (GOS) at discharge. Multivariable logistic regression models were used to assess the correlation between quantified hemorrhage volumes and the occurrence of complications and clinical outcomes (GOS) controlled for other variables such as age, sex, cardiovascular risk factors, clinical symptoms, and the modified Fisher scale. **Results**: A total of 82 patients (58.5% male, 54.8 ± 12.1 years) were enrolled. The median World Federation of Neurosurgical Societies (WFNS) score for all patients at admission was 1.0 (IQR 1.0–1.0). During the clinical course, hydrocephalus occurred in 29%, vasospasm in 14.6%, and DCI in 8.5% of all patients. Hemorrhage volume quantification was found to be the strongest independent predictor for hydrocephalus (OR 1.28; 95% CI 1.02–1.61; *p* = 0.032) and vasospasm (OR 1.25; 95% CI 1.07–1.46; *p* = 0.007) and showed a high predictive accuracy in ROC analyses (AUC = 0.77 and 0.76, respectively). Conversely, neither clinical parameters nor the modified Fisher scale were associated with these complications. A higher hemorrhage volume was also significantly correlated with a worse functional outcome (GOS; *β* = –0.07, CI: −0.12–−0.02, *p* = 0.021). **Conclusions:** In patients with pmSAH, the volumetric quantification of hemorrhage may be an adequate prognostic parameter regarding the occurrence of hydrocephalus and vasospasm. In addition, the quantitative assessment of hemorrhage volumes was strongly associated with clinical outcomes in these patients. Despite the generally benign nature of pmSAH, this imaging biomarker could improve individualized clinical management strategies and inform about the risk for the occurrence of complications.

## 1. Introduction

Perimesencephalic subarachnoid hemorrhage (pmSAH) is defined as non-aneurysmal subarachnoid hemorrhage, often localized anteriorly to the midbrain without expansion into the anterior interhemispheric or lateral Sylvian fissure and frank intraventricular or intraparenchymal hemorrhage [1]. They differ in their etiology, clinical course, and prognosis from aneurysmal SAH (aSAH). The prevalence of pmSAH is estimated to be in the range of 5–10% of all SAHs. It has been observed to manifest predominantly in middle-aged patients, with a higher propensity among males [2]. PmSAH is associated with a more favorable outcome, lower rates of rebleeding, and reduced mortality compared to aSAH [3]. Thus, pmSAH is often classified as a benign disease [4]. The pathophysiology of pmSAH is frequently unclear, and the source of bleeding may not be identified. Accordingly, venous and arterial causes are debated: The majority of studies suggest a non-arterial origin, presumably from venous or capillary leaks, potentially influenced by extrinsic forces (torsion, friction) or sudden intrathoracic pressure changes [2].

While pmSAH is generally considered benign, the occurrence of complications challenges this notion. These complications include the development of hydrocephalus, vasospasm, and delayed cerebral ischemia (DCI) [5,6]. Thus, despite the fact that the disease is generally associated with a favorable outcome, it is still important to be aware of its complications in order to avoid overlooking patients at risk.

It is therefore essential to identify patients who are at risk for complications in order to be able to intervene at an early stage. While existing clinical parameters such as the Glasgow Coma Scale (GCS) or the World Federation of Neurosurgical Societies (WFNS) score can have a certain predictive power for outcome assessment [7,8], they are also prone to be influenced by external factors (sedation, intubation, neurological variability). In contrast, imaging-based biomarkers can potentially enable a more objective assessment for the occurrence of complications.

Therefore, the aim of this study is to revalidate pmSAH as a benign entity and to identify prognostic factors that are associated with the occurrence of complications (hydrocephalus, vasospasm, DCI) and clinical outcome (Glasgow Outcome Scale, GOS) in order to improve early-risk stratification, with potential implications for clinical management, including monitoring strategies and treatment decisions. In addition, the study seeks to determine whether the imaging-based volumetric hemorrhage quantification of pmSAH on non-contrast head computed tomography may be a surrogate marker for the occurrence of complications and for clinical outcome.

## 2. Materials and Methods

### 2.1. Patient Selection and Study Design

This retrospective, single-center study included all consecutive patients presenting with pmSAH on initial brain imaging at a tertiary care academic medical center in Germany between 2010 and 2023. The study was conducted in accordance with the tenets of the Declaration of Helsinki (as revised in 2013), and the study was approved by the local ethics committee. Informed consent was not obtained from patients due to the retrospective nature of this study.

Patients eligible for this study were identified using the local radiology information system search mask with the keywords “perimesencephalic” or “prepontine” and “subarachnoid hemorrhage”. Patients were included if they were ≥18 years of age, of either sex, had a diagnosis of pmSAH based on non-contrast cranial CT (ncCT), and if complete clinical data were available in the electronic medical record (ORBIS), including GOS. Consequently, exclusion criteria were age < 18 years, poor-quality initial CT imaging that did not allow a reliable classification of the hemorrhage pattern, and incomplete clinical records regarding complications or outcome measures. Complications were documented as the presence or absence of hydrocephalus, vasospasm, and DCI during the clinical course.

Hydrocephalus was defined as ventricular enlargement on CT in combination with clinical signs of increased intracranial pressure (e.g., decreased consciousness or headache).

Vasospasm was defined as clinically relevant cerebral artery narrowing with delayed perfusion detected by CTA, CTP, DSA, or pathological findings at transcranial Doppler ultrasound and accompanied by a corresponding clinical symptoms, such as new focal neurological deficits or a drop in Glasgow Coma Scale (GCS) not explained by other causes (e.g., sedation, metabolic changes). Cases with isolated radiological or Doppler findings without clinical symptoms were not classified as vasospasms. This approach was chosen to identify clinically relevant vasospasm while avoiding overestimation based on asymptomatic imaging or flow changes.

DCI was defined as a new focal neurological deficit or a decrease in the level of consciousness lasting >1 h not explained by other causes (e.g., hydrocephalus, rebleeding, seizure, metabolic derangement) and/or a new ischemic lesion on follow-up imaging corresponding to clinical deterioration.

All patients were treated in accordance with our internal guidelines for patients presenting with SAH-like symptoms, including vasospasm prophylaxis with nimodipine.

### 2.2. Data Collection and Analysis

Baseline patient characteristics and clinical information were extracted from electronic medical records and examination records. The following variables were collected for each patient: demographics, including age and sex; clinical symptoms, including headache, neck stiffness, nausea, and vomiting; clinical scores, including the WFNS score; and imaging-based scores, including the modified Fisher scale [9,10]. Vascular risk factors included hypertension, smoking, diabetes, and alcohol consumption. Functional outcomes at hospital discharge were assessed using the GOS, a five-point scale that measures global neurological recovery, ranging from death (1) to good recovery (5). It provides a standardized assessment of functional status after brain injury and is widely used in the assessment of outcome after subarachnoid hemorrhage.

The diagnosis of pmSAH was made by ncCT at admission, follow-up imaging also included CT angiography (CTA), CT perfusion (CTP), and digital substraction angiography (DSA). The volumetric quantification of pmSAH was performed semi-automatically using the Siemens syngo.via software (version 05.01.0000.0070) on 3D ncCT images and was independently analyzed by two neuroradiologists with four and five years of experience in neuroimaging, blinded to clinical data and the study hypothesis. In the case of disagreement, consensus was reached through joint review and discussion. Neurological assessments as well as transcranial Doppler ultrasound for the assessment of vasospasms were carried out by trained neurosurgeons.

### 2.3. Statistical Analysis

Data are presented as mean ± standard deviation (SD) and median with interquartile range (IQR) for continuous variables, and as absolute numbers and percentages for categorical variables. Univariate and multivariable regression analyses were performed to evaluate potential predictors of complications (hydrocephalus, vasospasm, DCI) and functional outcome (GOS). Binary outcomes, including hydrocephalus, vasospasm, and DCI, were analyzed using logistic regression, providing odds ratios (ORs) with 95% confidence intervals (CIs) and corresponding *p*-values. Functional outcome measured by GOS was analyzed by linear regression, with *β* coefficients, standard errors (SEs), and 95% CIs. Although GOS is an ordinal variable, linear regression was used as an approximation to facilitate the interpretation of effect sizes. To adjust for potential confounders, multivariable logistic regression models were performed for each complication, including all collected variables as independent predictors, with results expressed as adjusted odds ratios (aOR) and 95% CIs. In addition, multivariable linear regression was used to assess independent predictors of GOS scores. Multicollinearity was assessed using variance inflation factors (VIFs), and adjustments were made as necessary.

To explore potential volume thresholds for clinical risk stratification, receiver operating characteristic (ROC) curve analyses were performed for the prediction of hydrocephalus and vasospasm. The Youden index (J = sensitivity + specificity − 1) was used to identify the optimal cut-off point for each outcome. The cut-off point corresponding to the maximum Youden index was chosen to reflect the best trade-off between sensitivity and specificity.

ROC curve analyses were also performed to assess the overall predictive performance of quantified hemorrhage volume. The area under the curve (AUC) was calculated to assess discriminatory ability, with higher AUC values indicating a better predictive performance. *p*-values < 0.05 were considered statistically significant. All statistical analyses were performed using GraphPad Prism (version 9.2.0, GraphPad Software Inc., San Diego, CA, USA) and SPSS Statistics version 29 (SPSS Inc., Chicago, IL, USA).

## 3. Results

### 3.1. Study Cohort

From 190 patients screened for this study, 82 patients with pmSAH (48 male, 34 female) and a mean age of 54.8 ± 12.1 years were enrolled. A total of 108 patients were excluded due to incorrect diagnosis, missing clinical data, or non-diagnostic quality of imaging (Figure S1). Most patients presented with a WFNS score of 1 (85.4%) at admission, while higher WFNS scores were less common (median: 1, IQR: 1–1). The most common symptom after pmSAH was headache (95.1%), followed by nausea (36.6%), neck stiffness (24.4%), and vomiting (21.9%). Cardiovascular risk factors included hypertension, which was present in 51.2% of patients, while smoking (9.8%), diabetes (8.5%), and alcohol consumption (3.7%) were less common. The modified Fisher scale distribution revealed that 34.1% of patients exhibited a score of 2, 35.4% exhibited a score of 3, and 30.5% exhibited a score of 4 (median: 3, IQR: 2–4). Notably, none of the patients demonstrated a modified Fisher score of 1. Of note, none of the patients exhibited frank intraventricular hemorrhage (IVH); however, minor blood accumulation or redistribution into ventricles led to modified Fisher grades 2 and 4. These IVH-like findings were graded visually by the neuroradiologists but were not included in the volumetric quantification, which was restricted to blood clots localized within the subarachnoid space. The median hemorrhage volume, as determined by semi-automated volumetry on ncCT, was 5.82 cm^3^ (IQR: 2.43–10.56 cm^3^).

**Figure 1:**
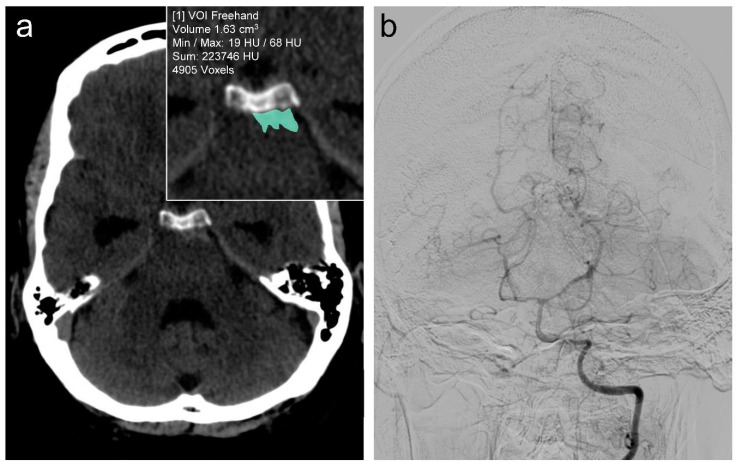
Exemplary patient case with pmSAH (modified Fisher scale 3) and the corresponding segmentation (green) in the magnification (**a**). The complications of hydrocephalus can be seen by the dilated temporal horns on non-contrast CT and the vasospasms in the posterior circulation (diagnostic angiogram via left vertebral artery indicating the segmental narrowing of basilar artery and posterior cerebral arteries on both sides, (**b**)).

Regarding the occurrence of complications, 29.3% of the pmSAH patients experienced hydrocephalus, 14.6% encountered vasospasm, and 8.5% exhibited DCI during the clinical course (exemplary case: Figure 1). Most patients exhibited a favorable outcome: The GOS distribution revealed that 90.2% of patients demonstrated a good recovery (GOS of 5), while 3.7% exhibited moderate disability (GOS of 4) and 6.1% exhibited severe disability (GOS of 3) (median: 5, IQR: 5–5). Detailed patient characteristics are shown in Table 1.

### 3.2. Analysis of Complications

To identify factors associated with the occurrence of complications, univariate and multivariate logistic regression analyses were performed for the presence of hydrocephalus, vasospasm, and DCI. In the univariate analysis, the assessed quantitative hemorrhage volume was strongly correlated with both hydrocephalus (*p* = 0.007, OR = 1.25, 95% CI: 1.07–1.46) and vasospasm (*p* = 0.009, OR = 1.19, 95% CI: 1.04–1.37), although no such correlation was found for DCI. Older age was weakly associated with hydrocephalus (*p* = 0.049, OR = 1.05, CI: 1.0–1.1), while no significant associations were found for cardiovascular risk factors, clinical symptoms, sex, or the Fisher scale (Table S1).

**Figure 2:**
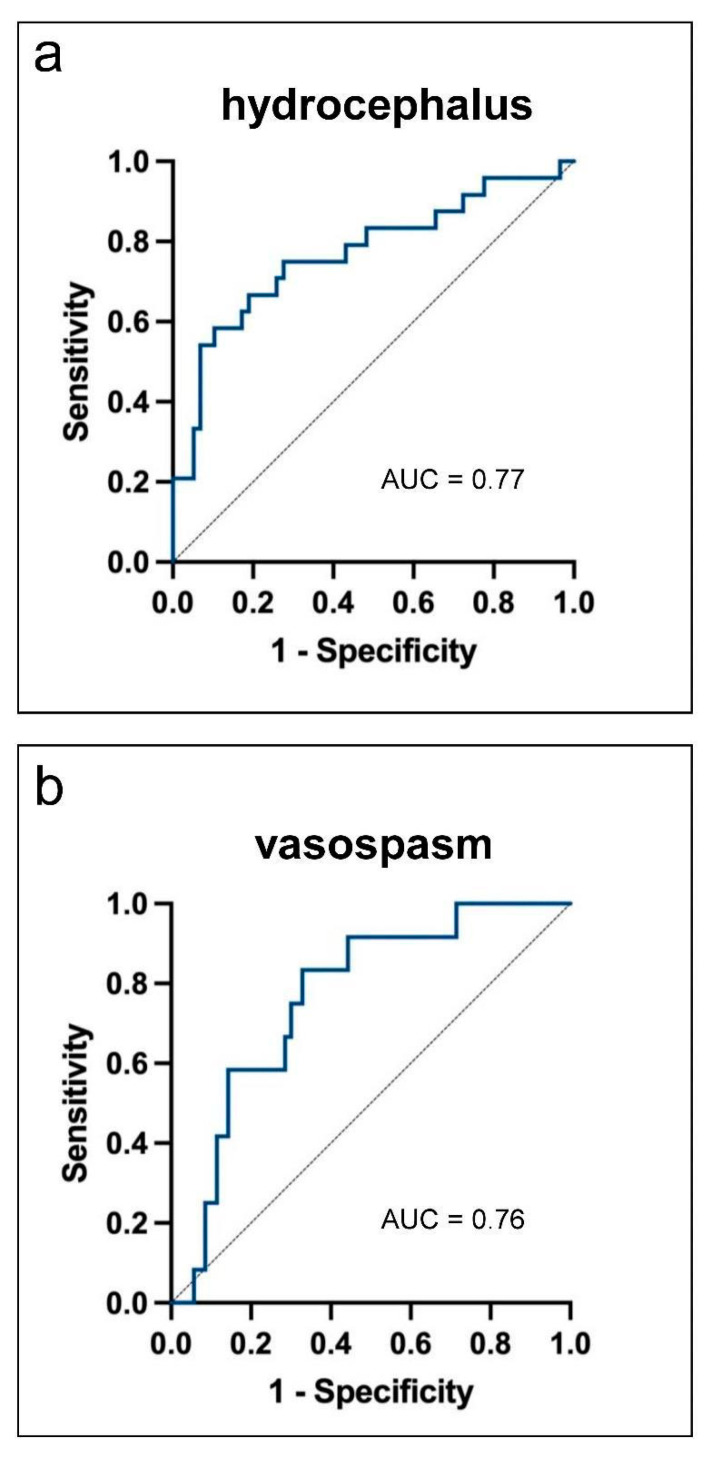
Receiver operating characteristic (ROC) curves illustrating the predictive performance of quantified blood volume for the occurrence of hydrocephalus (**a**) and vasospasm (**b**). AUC: area under the curve.

In multivariate regression analysis, hemorrhage volume remained the strongest independent factor associated with hydrocephalus (*p* = 0.032, OR = 1.28, 95% CI: 1.02–1.61) and vasospasm (*p* = 0.005, OR = 1.25, 95% CI: 1.07–1.46), whereas no variable showed a significant association with DCI (Table S2). The predictive performance of hemorrhage volume for hydrocephalus and vasospasm was further evaluated using ROC curves. The ROC curve for hydrocephalus (Figure 2a) showed an AUC of 0.77, indicating acceptable predictive performance. Similarly, the ROC curve for vasospasm (Figure 2b) yielded an AUC of 0.76, indicating a comparable predictive ability.

In addition, the Youden index was used to identify hemorrhage volume thresholds for clinical risk stratification. An optimal cut-off of 10.6 cm^3^ was identified for hydrocephalus (sensitivity 58%, specificity 90%) and 8.2 cm^3^ for vasospasm (sensitivity 83%, specificity 67%). These cut-offs may serve as exploratory reference points for future validation studies.

### 3.3. Analysis of Clinical Outcome

To assess functional outcomes, univariate and multivariable linear regression analyses were performed for the GOS. The univariate analysis revealed a significant association between the hemorrhage volume and GOS (*p* = 0.015, *β* = −0.05), suggesting that a higher hemorrhage volume is associated with poorer functional outcomes. In contrast, age exhibited a less pronounced association with GOS (*p* = 0.045, *β* = −0.02). No statistically significant associations were identified for cardiovascular risk factors, clinical symptoms, or the modified Fisher scale (Table S3).

In multivariable regression analysis, hemorrhage volume persisted as the most robust independent factor correlated with lower GOS scores (*p* = 0.021, *β* = −0.07), while age lost statistical significance after adjustment for covariates (Table S4). To ensure the validity of the multivariable model, multicollinearity was assessed, and all predictor variables, including hemorrhage volume, showed low VIF values, confirming the absence of relevant collinearity (Table S5).

## 4. Discussion

This study re-evaluates the benign nature of pmSAH and demonstrates the potential of an imaging-based hemorrhage volume quantification approach on ncCT for the prognostic risk assessment of complications and clinical outcomes. In our study, we assessed the occurrence of hydrocephalus, vasospasm, and DCI in 82 patients with pmSAH during their clinical follow-up. A significant correlation was identified between the occurrence of hydrocephalus and larger hemorrhage volumes, while no significant correlations with other clinical parameters were observed. Larger hemorrhage volumes were also independently associated with the development of vasospasm, regardless of other demographic and clinical parameters. No prognostic factors could be identified for DCI.

The demographics of our study align with those of other investigations on pmSAH, which predominantly included male patients in a middle-aged patient group [1,2,11,12]. In accordance with the findings of preceding studies, vascular risk factors such as arterial hypertension or smoking exhibited a rather heterogeneous distribution within the cohort and were therefore not found to be predictive for the subsequent clinical course [13,14,15]. This also applied to clinical symptoms such as headaches and nausea, which presumably describe initial pressure fluctuations and, therefore, occur in very high proportions, regardless of the occurrence of complications or respective clinical outcomes [16].

However, hemorrhage volume quantification on ncCT may provide a significant predictive value regarding the occurrence of hydrocephalus, likely due to cerebrospinal fluid (CSF) pathway obstruction or impaired resorption [17]. Although recent studies demonstrated an association between WFNS and the occurrence of hydrocephalus, they are subject to interobserver variability, subjective assessment, and patient non-compliance [18]. Conversely, imaging-based hemorrhage volume quantity can be regarded as a more objective and reproducible biomarker, independent of the patient’s clinical condition. After validation in larger multicenter cohorts, hemorrhage volume quantification may hold potential prognostic implications for clinical patient management, e.g., prophylactic external ventricular drain application when a certain bleeding volume threshold is exceeded.

Furthermore, the acquired volumes of perimesencephalic hemorrhage exhibited a significant correlation with the occurrence of vasospasm, which may be pathophysiologically attributable to an enhanced inflammatory response or endothelial irritation resulting from larger blood volumes [19]. In this case, it is important to note that clinical parameters such as the WFNS score were found to have no association with vasospasm risk.

The modified Fisher scale is a widely utilized tool for risk stratification in aSAH and for the development of vasospasm [10]. This scale incorporates the size expansion and intrusion into the ventricular system of SAHs. Although the modified Fisher scale is widely used in clinical practice and occasionally in research on pmSAH [3,20,21], its use appears to be a matter of routine rather than based on specific evidence or guideline recommendations. The present study underscores its limited capacity to predict complications. This may be attributable to the distinct bleeding patterns exhibited by pmSAH, which, in contrast to aSAH, is confined to the perimesencephalic and peripeduncular cisterns, as opposed to the diffuse basal cistern involvement observed in aSAH. This is further compounded by the potential absence of a significant subarachnoid blood burden in the convexity or Sylvian fissure, which plays a crucial role in the development of vasospasm in aSAH [22,23]. Furthermore, there may be differences in vascular activation and inflammatory response, as well as in CSF circulation and clot clearance in the peripeduncular cistern when compared to aSAH [24]. The present findings serve to reinforce the conclusion that the modified Fisher scale is not a suitable tool for the purposes of risk assessment in cases of pmSAH. Recently, the volumetric quantification of hemorrhage has also been successfully applied to predict complications in aSAH, identifying hemorrhage volume as an independent predictor of seizures and hydrocephalus [25]. Given the lack of reliable clinically established prognostic markers such as the modified Fisher scale in pmSAH, the volumetric quantification of hemorrhage volume may be even more valuable in predicting complications in this patient cohort.

The proportion of patients with pmSAH who developed vasospasm in this study is in good agreement with other studies, which estimate the incidence to be around 10% [26]. All available diagnostic modalities were used to assess vasospasm in this study, including CTA, CTP, DSA, and transcranial Doppler ultrasound, known for its particularly high specificity [27]. This comprehensive multimodal approach enhances the accuracy of vasospasm detection and is consistent with current recommendations for the thorough evaluation of patients with suspected pmSAH-related complications [28].

DCI describes clinical and radiological signs of cerebral ischemia, typically following an SAH. While vasospasm was initially considered to be the direct and sole cause of DCI, the current understanding favors a multifactorial genesis, including microvascular dysfunction, cortical spreading depression, inflammatory processes, and disturbances of autoregulatory mechanisms [1,29,30]. The incidence of DCI in this study was found to be low, consistent with the existing literature [1,31,32,33], and no significant prognostic indicators for the complication’s occurrence were identified. This finding is not unexpected given the multifactorial nature of its etiology. In addition, the limited number of DCI cases, the retrospective nature of the study and the complexity of overlapping pathophysiological mechanisms may have further reduced the statistical power to detect meaningful associations in our cohort.

As vasospasm is an important, but not the only factor contributing to DCI, it is apparent that parameters predictive of vasospasm may not necessarily be predictive of DCI. When looking at the complications of pmSAH, such as vasospasm and DCI, it is important to acknowledge potential iatrogenic triggers. Especially DSA, the prevailing gold standard when diagnosing pmSAH, can cause such complications through its manipulation of the endothelium [34]. However, these iatrogenic vasospasms occur immediately after the angiographic procedure [35] and were not included in this study.

Our study is limited by its retrospective nature. These include potential selection bias and lack of standardized follow-up imaging. In addition, the single-center design and limited sample size reduce statistical power and may limit the generalizability of our findings. Finally, although hemorrhage volume was semi-automatically segmented, interobserver variability and differences in scan quality may have influenced the accuracy of volume quantification. Due to the long study period and the use of different imaging systems, variability in scanner technology and imaging protocols cannot be completely excluded. Although only patients with adequate image quality were included, this heterogeneity remains a potential limitation of the study. Furthermore, due to the retrospective nature of the study, the exact time between symptom onset and first imaging could not be consistently determined. As a result, variability in time to imaging may have influenced volumetric quantification and clinical classification, especially regarding modified Fisher scale scoring. In addition, the high proportion of patients with a favorable outcome (GOS 5 in 90%) may have limited the ability of the model to detect predictors of poor outcome due to outcome homogeneity.

In summary, imaging-based hemorrhage quantification on ncCT was identified as a prognostic and objective biomarker for the occurrence of hydrocephalus and vasospasm in patients with pmSAH. In the future, hemorrhage volume quantification has the potential to serve as an image-based biomarker suitable for artificial intelligence applications, enabling automated risk stratification. However, to define cut-off volumes for risk stratification, larger cohorts in a multicentric validation approach are necessary. Furthermore, additional comparisons with aSAHs are required to enhance the comprehension of the divergent pathophysiology and risk assessment methodologies.

## 5. Conclusions

Despite the frequently benign genesis of pmSAHs, it is imperative to identify patients at risk for complications and poor clinical outcomes. Imaging-based hemorrhage volume quantification can be used as a non-invasive biomarker for the occurrence of hydrocephalus and vasospasm, and for the assessment of clinical outcomes. This approach demonstrates potential for facilitating personalized treatment decisions and subsequent patient management strategies.

## Figures and Tables

**Table 1:** Patient characteristics. DCI: delayed cerebral ischemia, GOS: Glasgow Outcome Scale, SD: standard deviation, WFNS: World Federation of Neurosurgeons.

Parameter	*n* (%)
Age (years, mean ± SD)	54.8 ± 12.1
Sex	
Male	48 (58.5%)
Female	34 (41.5%)
WFNS score	
1	69 (84.1%)
2	5 (6.1%)
3–5	8 (9.8%)
Modified Fisher scale	
1	0 (0%)
2	28 (34.1%)
3	29 (35.4%)
4	25 (30.5%)
Clinical symptoms	
Headache	78 (95.1%)
Neck Stiffness	20 (24.4%)
Nausea	30 (36.6%)
Vomiting	18 (22.0%)
Cardiovascular risk factors	
Hypertension	42 (51.2%)
Smoking	8 (9.8%)
Diabetes	7 (8.5%)
Alcohol	3 (3.7%)
Volumetry	
Hemorrhage volume (cm^3^, median (IQR))	5.18 (2.43–10.56)
Complications	
Hydrocephalus	24 (29.3%)
Vasospasm	12 (14.6%)
DCI	7 (8.5%)
Clinical outcome	
GOS	
5	74 (90.2%)
4	3 (3.7%)
1–3	5 (6.1%)

## Data Availability

The original contributions presented in this study are included in the article/Supplementary Materials. Further inquiries can be directed to the corresponding author.

## Supplementary Materials

The following supporting information can be downloaded at: https://www.mdpi.com/article/10.3390/biomedicines13051061/s1. Table S1: Univariate logistic regression results for the occurrence of complications; Table S2: Multivariate logistic regression results for the occurrence of complications; Table S3: Univariate linear regression results for the clinical outcome (GOS); Table S4: Multivariate linear regression results for the clinical outcome (GOS). Table S5: Variance inflation factor (VIF) values for all predictors included in the multivariable regression models. Figure S1: Flow chart of patient selection and analysis pathway.
