# Supplementary material for: Perimesencephalic Subarachnoid Hemorrhage Is Not Always a Benign Condition: Hemorrhage Volume as a Predictor for Complications and Clinical Outcome
Source: Biomedicines. 2025 Apr 27;13(5):1061. doi: 10.3390/biomedicines13051061 (PMC12109343; doi:10.3390/biomedicines13051061)
Supplement: Supplementary file 1 [file biomedicines-13-01061-s001.zip › biomedicines-3587247-supplementary.pdf]

Perimesencephalic subarachnoid hemorrhage is not always a benign condition: hemorrhage volume as a predictor for complications and clinical outcome (Supplementary Materials)

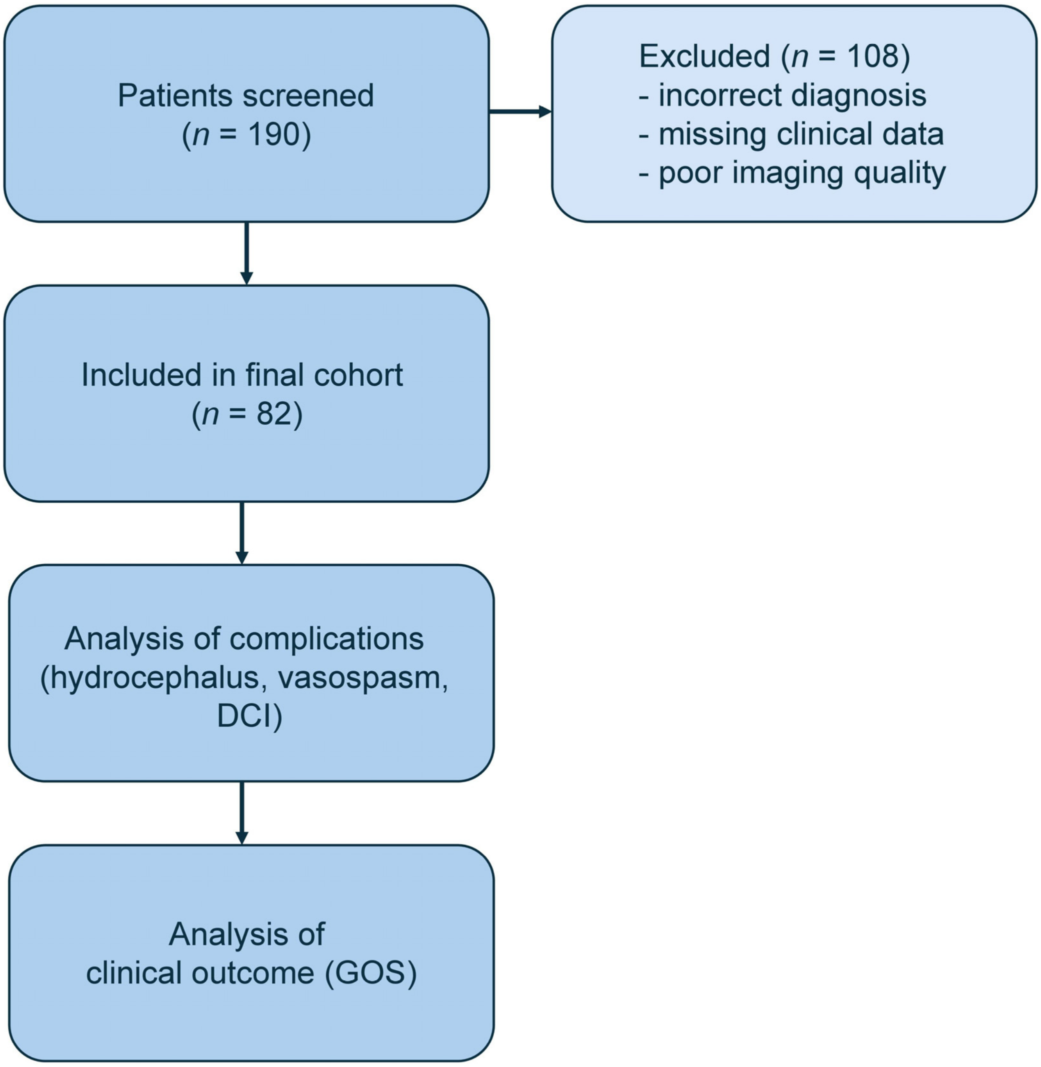

Supplementary Figure S1: Flow chart of patient selection and analysis pathway. *DCI: delayed cerebral ischemia, GOS: Glasgow Outcome Scale*

Variable	Hydrocephalus OR (95% CI)	*p*-value	Vasospasm OR (95% CI)	*p*-value	DCI OR (95% CI)	*p*-value
Hemorrhage volume	1.25 (1.07-1.46)	0.007	1.19 (1.04-1.37)	0.009	1.09 (0.87-1.37)	0.34
Age	1.05 (1.00-1.10)	0.049	1.02 (0.98-1.06)	0.41	1.01 (0.97-1.05)	0.48
Hypertension	1.06 (0.73–1.54)	0.76	1.03 (0.68–1.56)	0.82	1.07 (0.69–1.66)	0.78
Smoking	1.02 (0.45–2.31)	0.88	1.04 (0.49–2.22)	0.86	1.05 (0.50–2.20)	0.84
Diabetes	1.15 (0.59–2.25)	0.66	1.08 (0.55–2.12)	0.74	1.10 (0.56–2.14)	0.72
Alcohol	0.93 (0.41–2.13)	0.81	0.97 (0.45–2.07)	0.9	0.98 (0.46–2.09)	0.88
Headache	1.12 (0.76–1.68)	0.56	1.10 (0.74–1.63)	0.58	1.08 (0.72–1.61)	0.6
Neck stiffness	1.14 (0.75–1.73)	0.55	1.09 (0.71–1.68)	0.65	1.11 (0.72–1.71)	0.63
Nausea	1.08 (0.74–1.57)	0.62	1.02 (0.70–1.48)	0.82	1.03 (0.71–1.50)	0.81
Vomiting	0.97 (0.63–1.49)	0.72	0.99 (0.64–1.52)	0.91	1.00 (0.65–1.53)	0.95
Modified Fisher scale	1.10 (0.84–1.42)	0.44	1.05 (0.81–1.36)	0.6	1.06 (0.82–1.37)	0.56
WFNS score	1.15 (0.80–1.65)	0.42	1.12 (0.77–1.63)	0.45	1.09 (0.75–1.59)	0.48

Supplementary Table S1: Univariate logistic regression results for the occurrence of complications. *DCI: delayed cerebral ischemia, GOS: Glasgow Outcome Scale, SD: standard deviation, WFNS: World Federation of Neurosurgeons.*

Variable	Hydrocephalus OR (95% CI)	*p*-value	Vasospasm OR (95% CI)	*p*-value	DCI OR (95% CI)	*p*-value
Hemorrhage volume	1.28 (1.02–1.61)	0.032	1.25 (1.07–1.46)	0.005	1.07 (0.85–1.35)	0.38
Age	1.03 (0.98–1.08)	0.22	1.01 (0.96–1.05)	0.51	1.00 (0.96–1.04)	0.6
Hypertension	1.04 (0.72–1.51)	0.82	1.02 (0.67–1.54)	0.85	1.06 (0.68–1.63)	0.79
Smoking	1.01 (0.44–2.29)	0.94	1.03 (0.48–2.19)	0.89	1.04 (0.49–2.17)	0.87
Diabetes	1.12 (0.58–2.19)	0.7	1.06 (0.54–2.08)	0.78	1.09 (0.55–2.12)	0.73
Alcohol	0.91 (0.40–2.09)	0.83	0.96 (0.44–2.05)	0.92	0.97 (0.45–2.07)	0.89
Headache	1.10 (0.74–1.65)	0.57	1.09 (0.73–1.61)	0.59	1.07 (0.71–1.59)	0.61
Neck stiffness	1.12 (0.73–1.71)	0.59	1.07 (0.69–1.65)	0.69	1.10 (0.71–1.68)	0.66
Nausea	1.06 (0.72–1.55)	0.75	1.01 (0.69–1.47)	0.86	1.02 (0.70–1.48)	0.83
Vomiting	0.95 (0.62–1.45)	0.8	0.98 (0.63–1.50)	0.89	0.99 (0.64–1.52)	0.92
Modified Fisher scale	1.08 (0.82–1.40)	0.5	1.04 (0.80–1.34)	0.63	1.05 (0.81–1.36)	0.58
WFNS score	1.13 (0.78–1.62)	0.44	1.11 (0.76–1.61)	0.46	1.08 (0.74–1.58)	0.5

Supplementary Table S2: Multivariate logistic regression results for the occurrence of complications.
DCI: delayed cerebral ischemia, GOS: Glasgow Outcome Scale, OR: odds ratio, SE: standard error, WFNS: World Federation of Neurosurgeons.

Variable	β (95% CI)	SE	*p*-value
Hemorrhage volume	-0.05 (-0.09 – -0.01)	0.02	0.015
Age	-0.02 (-0.04 – -0.00)	0.01	0.045
Hypertension	-0.01 (-0.05 – 0.03)	0.03	0.62
Smoking	0.00 (-0.07 – 0.07)	0.04	0.98
Diabetes	-0.01 (-0.09 – 0.06)	0.03	0.75
Alcohol	0.00 (-0.12 – 0.12)	0.05	0.99
Headache	-0.01 (-0.07 – 0.05)	0.03	0.72
Neck stiffness	-0.01 (-0.06 – 0.04)	0.02	0.78
Nausea	-0.01 (-0.07 – 0.05)	0.03	0.71
Vomiting	-0.00 (-0.06 – 0.06)	0.04	0.94
Modified Fisher scale	-0.02 (-0.06 – 0.02)	0.02	0.3
WFNS score	-0.03 (-0.08 – 0.02)	0.03	0.25

Supplementary Table S3: Univariate linear regression results for the clinical outcome (GOS). *GOS: Glasgow Outcome Scale, SE: standard error, WFNS: World Federation of Neurosurgeons.*

Variable	β (95% CI)	SE	*p*-value
Hemorrhage volume	-0.07 (-0.12 – -0.02)	0.02	0.021
Age	-0.01 (-0.03 – 0.01)	0.01	0.19
Hypertension	-0.01 (-0.05 – 0.03)	0.03	0.61
Smoking	0.01 (-0.06 – 0.08)	0.04	0.92
Diabetes	-0.01 (-0.08 – 0.06)	0.03	0.79
Alcohol	0.00 (-0.11 – 0.11)	0.05	0.97
Headache	-0.01 (-0.07 – 0.05)	0.03	0.7
Neck stiffness	-0.01 (-0.05 – 0.04)	0.02	0.75
Nausea	-0.01 (-0.07 – 0.05)	0.03	0.69
Vomiting	0.00 (-0.06 – 0.06)	0.04	0.96
Modified Fisher scale	-0.02 (-0.06 – 0.02)	0.02	0.32
WFNS score	-0.03 (-0.07 – 0.01)	0.03	0.28

Supplementary Table S4: Multivariate linear regression results for the clinical outcome (GOS). *GOS: Glasgow Outcome Scale, SE: standard error, WFNS: World Federation of Neurosurgeons.*

Variable	VIF
Hemorrhage volume	1.71
Age	1.69
Hypertension	1.49
Smoking	1.13
Diabetes	1.22
Alcohol	1.41
Headache	1.11
Neck stiffness	1.16
Nausea	1.93
Vomiting	1.82
Modified Fisher scale	1.65
WFNS score	1.54

Supplementary Table S5: Variance inflation factor (VIF) values for all predictors included in the multivariable regression models. All VIF values were < 2, indicating no relevant multicollinearity among predictors and supporting the stability of the regression estimates. *WFNS: World Federation of Neurosurgeons.*
